# Conceptual tensions and practical trade-offs in tailoring implementation interventions

**DOI:** 10.3389/frhs.2022.974095

**Published:** 2022-11-17

**Authors:** Sheena M. McHugh, Fiona Riordan, Geoff M. Curran, Cara C. Lewis, Luke Wolfenden, Justin Presseau, Rebecca Lengnick-Hall, Byron J. Powell

**Affiliations:** ^1^School of Public Health, University College Cork, Cork, Ireland; ^2^Department of Pharmacy Practice and Psychiatry, University of Arkansas for Medical Sciences, Little Rock, AR, United States; ^3^MacColl Center for Health Care Innovation, Kaiser Permanente Washington Health Research Institute, Seattle, WA, United States; ^4^College of Medicine, Health and Wellbeing, The University of Newcastle, Callaghan, NSW, Australia; ^5^Clinical Epidemiology, Ottawa Hospital Research Institute, Ottawa, ON, Canada; ^6^Center for Mental Health Services Research, Brown School, Washington University in St. Louis, St. Louis, MO, United States; ^7^Center for Dissemination and Implementation, Institute for Public Health, Washington University in St. Louis, St. Louis, MO, United States; ^8^Division of Infectious Diseases, John T. Milliken Department of Medicine, School of Medicine, Washington University in St. Louis, St. Louis, MO, United States

**Keywords:** tailoring, implementation science, concept, commentary, chronic disease

## Abstract

Tailored interventions have been shown to be effective and tailoring is a popular process with intuitive appeal for researchers and practitioners. However, the concept and process are ill-defined in implementation science. Descriptions of how tailoring has been applied in practice are often absent or insufficient in detail. This lack of transparency makes it difficult to synthesize and replicate efforts. It also hides the trade-offs for researchers and practitioners that are inherent in the process. In this article we juxtapose the growing prominence of tailoring with four key questions surrounding the process. Specifically, we ask: (1) what constitutes tailoring and when does it begin and end?; (2) how is it expected to work?; (3) who and what does the tailoring process involve?; and (4) how should tailoring be evaluated? We discuss these questions as a call to action for better reporting and further research to bring clarity, consistency, and coherence to tailoring, a key process in implementation science.

## Introduction

Judicious use of implementation strategies can enhance the adoption (1), implementation (2), and impact of evidence-based interventions (EBIs) (3). Implementation strategies, “methods or techniques used to enhance the adoption, implementation, and sustainment of a clinical program or practice” (4), are often multifaceted complex interventions, involving combinations of discrete strategies (e.g., audit and feedback, reminders, education/training, etc.). There are a number of published taxonomies describing implementation strategies (5–8), but with lots of options from which to choose and multiple ways of operationalizing implementation strategies, the task of selecting and operationalizing strategies is challenging. In addition, practitioners and organizations face different implementation barriers and enablers (determinants) depending on the local context. And, local factors can affect the selection of strategies, for example, constraints, incentives, and pressures in the local environment. While tailoring is a suggested approach to select and modify strategies to address these local determinants (9–12), what constitutes tailoring is ambiguous, and the best way to ensure strategies are capable of addressing contextual needs is not yet known.

Although descriptions and applications of tailoring vary, tailoring has generally been described as a prospective process for selecting and modifying strategies to address determinants (9). Tailoring strategies is part of an effort to exact change through determinant-strategy alignment and increase implementation success (9, 10). The first step involves identifying contextual factors that influence implementation of the particular EBI in a given setting. The second step involves prioritizing certain determinants and matching and modifying implementation strategies to address those determinants, before applying those strategies.

**Figure 1:**
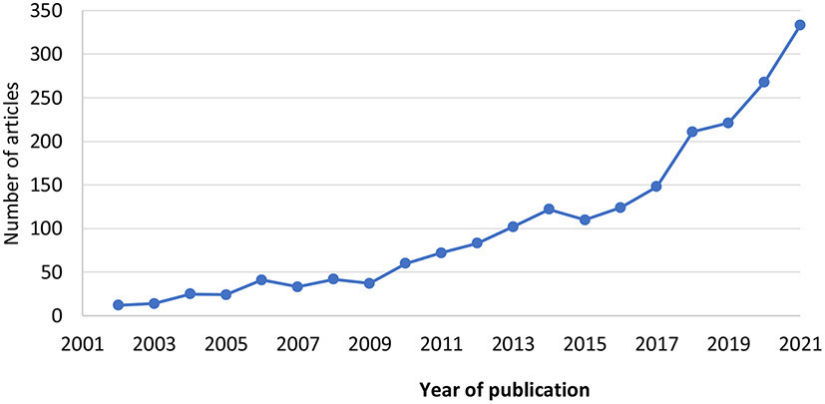
Papers referencing terms for tailoring and implementation and healthcare from 2002 to 2022, published in MEDLINE, based on searching titles and abstracts and subject headings. The search was run May 5th, 2022 with no limits on publication type or language.

There is some evidence to suggest the output of this process is effective. A Cochrane review of the effectiveness of tailored implementation strategies identified 32 trials, and reported a small to moderate effect in those comparing a tailored to a non-tailored strategy [Odds Ratio = 1.79 (95% CI 1.06–3.01, *p* = 0.033)] (9). Since December 2014, when the most recent search for the Cochrane review was published, there appears to be substantial growth in the number of studies focusing on tailored implementation strategies. We conducted an electronic search for key terms (“tailor^*^” and terms for strategy or intervention and implementation and healthcare) in title and abstracts along with relevant subject headings in MEDLINE (13). In the last 20 years, there has been a clear upward trend in articles referring to tailoring (Figure 1), with 1,722 articles published 2012–2021 compared to 443 articles in the 10 years (2002–2011) previous.

The 2015 Cochrane review identified substantial variation in tailoring approaches, a lack of detail about how the process was conducted, insufficient rationale underpinning the tailoring process, and no assessment of the relative costs of tailored strategies. The authors called for more description of the methods for selecting and designing tailored strategies including comparisons of different methods, and RCTs to identify tailoring approaches more likely to lead to behavior change and to determine cost-effectiveness.

To this end, we believe the field needs conceptual grounding on which to build empirical evidence. We propose four interrelated questions about the tailoring process that need to be addressed to advance the understanding and application of tailoring in research and practice: (1) what constitutes tailoring; and when does it begin and end; (2) how is it expected to work; (3) who and what does the tailoring process involve; (4) and, how should tailoring be evaluated?

### What is tailoring and when does it begin and end?

There is no consensus on the definition of tailoring. Table 1 illustrates variable definitions across widely used frameworks and reporting guidelines. Definitions vary in terms of the purpose of tailoring (is the aim of tailoring to prospectively and purposefully develop/select strategies to address contextual factors, or iteratively adapt strategies as context and needs change, or both), and phase of implementation (is tailoring part of a strategy development process or is it a component within a broader multifaceted implementation strategy or both). Both can be understood as a question of timing, that is, when does tailoring begin and end. For example, the term tailoring has been used to describe modifications or personalization made to strategies *in advance* to fit with different population subgroups (14–16). In contrast, it has also been used to describe adaptations made to implementation strategies throughout an implementation effort (11). The Framework for Reporting Adaptations and Modifications to Evidence-based Implementation Strategies (FRAME-IS) refers to the modification of strategies after deployment as ongoing tailoring (17). For some, tailoring is considered solely an initial design process before deployment of an implementation strategy, while for others it is an iterative process that continues during strategy deployment as implementation challenges arise.

**Table 1:** Definitions of “tailoring” in select frameworks and reporting guidelines.

**Source**	**Definition of tailoring**
Expert recommendations for implementing change (ERIC) (5)	“Tailor strategies”: Tailor the implementation strategies to address barriers and leverage facilitators that were identified through earlier data collection.
Knowledge to action (KTA) framework (18)	Describes process of knowledge translation including: identify the implementation problem the know/do gap, create or identify and select knowledge relevant to, **adapt knowledge to local context**, assess barriers/facilitators to knowledge use, **select, tailor, and implement interventions to promote knowledge use**, monitor, evaluate and sustain knowledge use. Refers to selecting and tailoring interventions to the identified barriers and audiences (18).
Template for intervention description and replication (TIDieR) (19)	Includes tailoring as part of the template for describing interventions. It refers to **tailoring as whether and how the intervention is** ***planned*** **to be personalized, adapted**.
Framework for reporting adaptations and modifications to evidence-based implementation strategies (FRAME-IS) (17)	Refers to the modification of strategies **after deployment** as ongoing tailoring
Standards for reporting implementation studies (StaRI)	Refers to **fidelity/adaptation**; Fidelity to implementation strategy as planned and adaptation to suit context and preferences

Bold text highlights the main part of the definition relating to tailoring.

In terms of *phase*, to add to this complexity, tailoring itself may be part of an implementation strategy being deployed to support an EBI (20–23). For example, in the Collaborative Organizational Approach to Selecting and Tailoring Implementation Strategies (COAST-IS) approach organizations were coached to use Intervention Mapping (also referred to as Implementation Mapping) to tailor implementation strategies to local contextual barriers. These challenges with defining tailoring are not unique to implementation science. The behavioral science field also grapples with clarifying the distinction between interventions customized at the individual level (24, 25), and targeted interventions which are modified in advance based on selected characteristics of population subgroups (26). It is important to note, that while we distinguish tailoring here by purpose and phase, tailoring could be both prospective (proactive) *and* iterative throughout the implementation process.

### How is tailoring expected to work?

If the determinants of practice that can influence implementation are identified, and strategies are then selected and deployed to address the determinants, it would seem reasonable to expect greater implementation success. While there are assumptions about how tailoring is intended to work, the underpinning logic has not been made explicit. This theorizing could point toward the circumstances in which tailoring would be most effective (10) or guide the selection of indicators of “success”.

Using concept analysis (27), researchers defined the attributes of tailoring, providing some indication of how tailoring is expected to lead to better implementation. The results suggest that tailoring (1) can have two targets: evidence (tailoring, for example, best practice guidelines to local context) and context (identifying and addressing determinants related to context, for example, organizational resources, structures, culture, personnel); (2) involves explorative, consensus building methods; (3) involves collaboration and engagement with the end users to raise awareness and obtain buy-in; (4) aims to create a fit between the tailored strategy and organizational context; (5) is an active and iterative approach; and (6) should address the challenges of ensuring fidelity to the evidence. Potential mechanisms of action leading from tailoring to changes in implementation outcomes could include (a) raising awareness of problems, (b) building stakeholder consensus, (c) generating buy-in or acceptance, or (d) creating greater coherence between the context and implementation strategy. These mechanisms may be moderated by features of the tailoring process such as the number of steps and the extent to which to which stakeholders are represented and involved, while time and resources for tailoring, along with knowledge of the local context, may act as preconditions.

### Who and what does the tailoring process involve?

While acknowledging the potential for reactive tailoring, there is still a temporality or sequence inherent in tailoring. Strategy selection must follow some identification or acknowledgment of the determinants of practice. Studies may delineate some or all of the following steps: (a) the identification of implementation determinants, (b) prioritization of determinants, (c) selection of strategies by matching to determinants (28–31), and (d) execution of strategies. This step may involve operationalizing each strategy (deciding who does what, when and where and developing materials) [e.g., (20)], or it may extend to enacting the strategy (32). Research suggests that while many strategies are planned as a result of tailoring, fewer are executed (32). With stakeholder involvement, this step may provide even more opportunities to realize the holistic effects of the tailoring process, triggering mechanisms such as sense-making and generating buy-in.

Previous research has tended to focus on the earlier steps of tailoring. The Tailored Implementation for Chronic Disease (TICD) study (28), found that a combination of methods (e.g., brainstorming, and structured interviews with patients and health care professionals) should be used to identify a larger number of determinants. However, they reported little effect of tailored strategies on primary and secondary outcomes, proposing lack of fidelity to the tailored strategies, incomplete identification of determinants, short follow-up period, and insufficient matching of strategies with determinants, among the possible reasons for the lack of effect.

The mismatch between implementation determinants and the functions of the proposed strategies is indicative of the problems associated with the lack of systematic and well-described methods at the prioritization and selection stage (33, 34). Studies have used a variety of methods to match strategies to determinants using popular tools such as the CFIR-ERIC matching tool (35), and the Theoretical Domains Framework and Behavior Change Wheel (BCW) (36, 37). In a study by Schroeck et al. (38) researchers “cross walked” all possible ERIC strategies against determinants that were coded using the TICD framework. Similarly, Becker-Haimes et al. (39) used both ERIC and the BCW to match strategies to determinants. The COAST-IS project adopted a participatory approach whereby researchers coached local stakeholders to use a particular method to tailor implementation strategies (Intervention Mapping) (20). It is important to acknowledge that matching is done largely in the absence of empirical data on the causal effects of strategies in addressing specific barriers. Audit and feedback (A&F) may be considered one exception as evidence on how this strategy works is growing (40–43). Work is underway to explore and test the mechanisms of action of other strategies which will inform determinant-strategy matching (44–47).

In summary, less is known about the latter steps of tailoring, prioritizing determinants, and matching and selecting strategies, and how these decisions are made. While there is value in these steps continuing to be “creative” with in-built flexibility, implementation researchers have called for more systematic (9, 10, 33), innovative (21), and transparent approaches to prioritizing determinants and selecting strategies, the application of which in specific sites/settings is not always clear (28). The lack of transparency around how or why decisions are made during tailoring limits the potential to fully evaluate the process and its output, the tailored strategy.

#### Role of theory, evidence, and stakeholders

Theory, evidence, and stakeholders' perspectives are proposed as important ingredients in the tailoring process (10), but there are practical issues when trying to integrate these elements. These challenges have been highlighted in relation to the development of complex health interventions more generally (48) where end-user engagement and theory are also considered critical parts of the process (49).

The way in which stakeholder contributions and knowledge should be combined with evidence and theory are unclear, particularly if these contradict one another (50). Based on their process evaluation of five tailored programs to improve implementation of different chronic disease interventions in primary care, Jager et al. suggested some important determinants may not have been addressed as the health care professionals involved assumed they were not relevant or modifiable (34). Similarly, as part of a consensus process to develop a strategy to support uptake of diabetic retinopathy screening, O'Mahony et al. found that stakeholders (professionals and people with diabetes) had different perspectives on which behavior change techniques were feasible and acceptable to include in the final strategy (51). This may be indicative of the differences between stakeholder assumptions about what should be prioritized. It also suggests that the process and output of tailoring could vary depending on *who* is driving the exercise.

The criteria used to prioritize determinants and select strategies should be considered carefully as they influence the resulting strategy. Decisions made when prioritizing determinants often appear to be based on stakeholder perceptions of the modifiability and importance of determinants, while strategy selection is often based on perceived feasibility and impact of strategies (29, 31, 34). Wensing and Grol suggest that organizational and system-related problems can be ignored in favor of individual, educational and psychological approaches (50). There have been calls for consideration of a broader range of criteria when prioritizing determinants and selecting strategies (52). Moreover, different criteria may be important at each stage. For example, criteria, such as criticality (how likely it is that a determinant affects an implementation outcome), chronicity (how frequently a determinant occurs or persists), and ubiquity (how pervasive a determinant is,) may be valuable to guide the prioritization of determinants (52). It is important that researchers consider the range of criteria that could be used and how these criteria can be assessed and reconciled.

As suggested above, researchers and health system stakeholders have different expertise, assumptions and perspectives that may shape the process of tailoring. How and when they are involved may in theory also influence the process and final output (29, 31, 34). Some studies have compared different methods to involve stakeholders in strategy development. As part of the TICD study, Krause et al. found a greater number of plausibly important determinants were identified by health care professionals (*via* brainstorming or interviews) than by patients (*via* interviews). Recent research to develop implementation strategies (39) compared a more traditional qualitative approach (observations and interviews) with rapid survey-based approach (innovation tournament). There is a general belief in the value of stakeholder involvement in tailoring, and a theory that how they are involved will influence the outcomes of tailoring. However, there is little evidence to date on the effect of different approaches, or *how* involvement can best be approached in different contexts and aligned with stakeholder preferences, pointing to the need to better describe, understand and evaluate methods to involve stakeholders in strategy development (50).

Being more explicit about who is driving tailoring, with whom and for whom will inform thinking about how tailoring is expected to work, what success looks like and how it can be evaluated. There are several examples of tailoring driven primarily by researchers and involving health system stakeholders (e.g., health professionals and managers) at various stages (38, 39, 53). In contrast, other tailoring studies focus on equipping health system stakeholders to drive tailoring within their organization [such as COAST-IS (20) and ImpleMentAll (11)]. It may be that some steps are more appropriately driven by researchers or by local health system stakeholders. Depending on the service context (i.e., staffing, local expertise, and funding), the end goal may be for healthcare practitioners (local stakeholders) to work independently to tailor implementation strategies as they need to, *or* for researchers and healthcare practitioners to work together, to design tailored implementation strategies that can ultimately enhance patient care. It is important to consider the skills that may be required to facilitate tailoring in practice. As part of work to identify core competencies among individuals working in implementation science, Metz et al., suggest that implementers need skills to be able to address power imbalances, work collaboratively, facilitate knowledge exchange between stakeholders (brokering) to ensure different perspectives are incorporated, and use co-design tools and resources with a commitment to participatory processes (54). Similarly, Gonzales et al. (55) in setting out a framework for training healthcare professionals in implementation science, suggests the importance of being skilled in developing relationships with those in the local context, and integrating diverse perspectives when developing implementation strategies.

#### Feasibility and acceptability of the process

Finally, approaches to tailoring will likely vary in terms of their feasibility, acceptability, level of involvement and research rigor depending on the demands and constraints within a project, organization, or the broader health system. There are concerns (31) that a divide is growing between best practices from implementation research and what is actually happening and feasible, in practice, for stakeholders (56). For example, there are technology and resource requirements and specific expertise required for some tailoring methods that may prohibit their independent and sustained application in practice settings. There are some examples to generate scalable, accessible approaches, for instance, the ImpleMentAll project's self-directed toolkit for tailoring (11), the CFIR-ERIC Implementation Strategy Matching Tool (35), and the Behavior Change Taxonomy Theory and Techniques Tool (57). The number of steps, which stakeholders are involved and their role, and choice of methods are a series of decisions likely influenced by the resources available for tailoring within a setting. Exploring the ways in which tailoring is conducted in different health system contexts could inform decisions about which approaches are optimal and for whom in what circumstances.

### How should the tailoring process be evaluated?

Given practical complexities in tailoring, Baker et al. called for research on the “most promising” methods by comparing prioritization and selection methods to identify those most likely to lead to “successful” tailoring. However, the issue is complicated by a lack of clarity around how the “success” of tailoring should be determined. Tailoring is typically evaluated using a summative assessment of the effectiveness of the tailored strategy in improving implementation. But there is a distinction between evaluating the process of tailoring and evaluating the outcome, the strategy (58). Often evaluative studies of implementation strategies use outcome measures like clinical behaviors (50). The use of more pragmatic (and interim) measures like acceptability, feasibility, compatibility with existing workflows and perceived usefulness has also come to the fore (59, 60). Without interim measures of the tailoring process, establishing causality is difficult. We lack measures but also designs to compare methods of tailoring. Newer designs like Sequential Multiple Assignment Randomized Trials (SMART) may offer possibilities to further our understanding of the tailoring process, albeit within constraints. With SMART, sites are randomized to adaptations of an implementation strategy based on pre-specified decision rules, allowing the effect of these decisions (strategy modifications), whether they are ultimately beneficial or not, to be isolated (61).

When establishing causality, it is important to identify proximal and distal outcomes that the intervention (in this case, the tailoring process) is theorized to impact. Where an underlying theory and mechanism of action is proposed, there is potential to evaluate secondary outcomes. However, without a clear articulation of the mechanism of action, and the mediators and moderators of tailoring, it is difficult to determine suitable proximal outcomes to evaluate the success of the tailoring process (44). We have yet to determine what proximal measures can and have been used (29). When assessing methods to identify determinants as part of the Tailored Implementation in Chronic Disease study, Huntink et al. (62) found it difficult to assess the “validity” (“correctness” or “fit”) of the strategies generated through group interviews with end-users as there was no reference point for this assessment. While an assessment of fit is not built-in to the tailoring process, theoretical coherence, that is the “match” between intervention components (behavior change techniques) and determinant domains, has been used as an indicator of the quality of the behavior change intervention development process (63). This indicator would align with a potential mechanism by which tailoring could work; that is creating coherence between the context and the strategy. Aligning with other potential mechanisms mentioned earlier, assessing stakeholder engagement and “buy-in” may represent other possible ways to judge the “success” of tailoring (64).

## Discussion

Despite more studies focused on tailoring, there is a lack of clarity about if and how tailoring is expected to, and actually works (or fails), in different contexts to achieve successful implementation. Descriptions of *how* tailoring has been applied in practice are often absent or insufficient in detail, including the contexts in which it has been used, at what stages and who has been involved in the process (10). In summary, we believe there are four main questions which should be asked of tailoring to move toward great clarity in its definition, operationalization, underpinning logic, and evaluation: (1) What constitutes tailoring and when does it begin and end?; (2) How is it expected to work?; (3) What does the tailoring process involve?; and (4) How should tailoring be evaluated? To advance research and practice, we propose there is a need for international consensus on the core components and functions of tailoring in the context of implementation science *and* consensus on the elements of tailoring which need to be systematically reported.

In terms of clarifying the core components and functions of tailoring, we feel that with the increasing focus on tailoring, now is an opportune time to synthesize existing examples to see how tailoring is defined and applied. We are engaged in an ongoing scoping review to bring together the available evidence (13), including how tailoring is conceptualized, operationalized, and evaluated, to shed light on some of the hidden trade-offs for researchers and practitioners that are inherent in the process. There may be value in learning from efforts to systematize and categorize different approaches to developing complex interventions (49, 65).

In terms of reporting, first, we suggest researchers and practitioners in the field articulate their adopted definition of tailoring, reporting both the intention (whether a one-off design exercise or ongoing iterative process) and phase of tailoring (used to develop/refine strategies and/or part of a larger meta-strategy). Second, to develop our understanding of the tailoring process, we suggest the approaches used to prioritize determinants, match them to strategies, and select strategies, should also be clearly articulated. Specifically, researchers and practitioners should report whether and how evidence and theory were drawn on, detail the methods used to involve stakeholders, and outline the types and range of criteria on which decisions were based. Lastly, reflections on the feasibility and acceptability of the tailoring process from the perspective of stakeholders with reference to their context should also be reported. There is dearth of evidence on the feasibility, acceptability, effectiveness, and cost of different tailoring approaches to inform and guide researchers and practitioners undertaking tailoring in different contexts (10). To partly address this gap, we are conducting case studies in the Irish health system to explore the prioritization and selection stage and using a multiple case study approach to compare stakeholder's experiences of tailoring implementation for different health care priorities and in different health service settings. To improve reporting of the tailoring process, researchers could draw from items in the guidelines for reporting the development and evaluation of complex interventions in health care (66), the TIDieR guideline (19) or the recommendations for reporting implementation strategies (67). We also suggest that researchers be explicit about how and why the tailored implementation strategies may work together synergistically (68, 69), what they consider the core and peripheral components, and the mechanisms through which they expect their strategies to operate (44, 46, 47, 70). During the tailoring process is an opportune time to discuss these hypotheses with different stakeholders.

We believe highlighting the lack of transparency in the tailoring process and some of the key questions is a valuable first step. We propose that achieving consensus on the core components and functions of tailoring *and* the elements of tailoring which need to be systematically reported is key to aid implementation researchers in the future synthesis and replication of tailoring efforts, furthering the development of the field. Working together as a community to pay attention to these aspects when reporting on tailoring, along with undertaking new research to specifically explore and address these questions, will bring clarity, consistency, and coherence to tailoring, a key process in implementation science.

## Data availability statement

The original contributions presented in the study are included in the article/supplementary material, further inquiries can be directed to the corresponding author.

## Author contributions

SM, CL, GC, JP, and BP conceived of the paper. SM and FR prepared drafts of the paper. All authors reviewed drafts of the paper and approved the final version.

## Funding

SM and FR were funded through a Health Research Board Research Leader Award (RL-2020-004). LW was supported by a NHMRC Investigator Grant (APP1197022), and receives infrastructure support from the Hunter Medical Research Institute. GC was supported by the Translational Research Institute (TRI), UL1 TR003107, through the (US) National Center for Advancing Translational Sciences of the National Institutes of Health (NIH). CL was supported by P50MH126219, R01CA262325, P50CA244432, and R13HS025632. RL-H was supported by NIMH P50MH113662. BP was supported by R13HS025632, R01CA262325, R01DA047876, P50CA19006, R01HL157255, and R25MH080916.

## Conflict of interest

The authors declare that the research was conducted in the absence of any commercial or financial relationships that could be construed as a potential conflict of interest.

## Publisher's note

All claims expressed in this article are solely those of the authors and do not necessarily represent those of their affiliated organizations, or those of the publisher, the editors and the reviewers. Any product that may be evaluated in this article, or claim that may be made by its manufacturer, is not guaranteed or endorsed by the publisher.
